# PLAC8 Overexpression Promotes Lung Cancer Cell Growth via Wnt/*β*-Catenin Signaling

**DOI:** 10.1155/2022/8854196

**Published:** 2022-04-22

**Authors:** Wei Chen, Junlu Wu, Weiwei Wang, Li Yu, Xianghuai Xu

**Affiliations:** ^1^Department of Pulmonary and Critical Care Medicine, Tongji Hospital, School of Medicine, Tongji University, Shanghai 200065, China; ^2^Department of Laboratory Medicine, Tongji Hospital, School of Medicine, Tongji University, Shanghai 200065, China; ^3^Department of Pathology, Tinghu People's Hospital of Yancheng City, Yancheng 224001, China

## Abstract

The PLAC8 expression in lung cancer tissues and in vitro grown lung cancer cells, as well as the involvement of the Wnt/*β*-Catenin signaling pathway, was investigated in this process. PLAC8 protein expression in human lung cancer tissues and lung tumor cells of different strains was discovered using immunohistochemistry staining and Western blot, respectively. Animal models of PLAC8 overexpression and knockdown were created using lentivirus. The development in tumor tissue was seen both in vitro and vivo. The Wnt/*β*-Catenin signaling pathway played an important part in this process, as shown by the dual luciferase reporter gene system. PLAC8 expression was elevated in lung cancer tissues and plasma and decreased in plasma after lung tumor resection. PLAC8 upregulation promotes cell proliferation in vivo and in vitro, while PLAC8 downregulation inhibits cell viability and proliferation. The results of the dual luciferase reporter gene system suggest that PLAC8 can significantly activate the Wnt/*β*-Catenin signaling pathway in cells and can conduct signaling through it. A potential treatment targeting the prognosis of lung cancer patients may be PLAC8 overexpression, which promotes the lung cancer cell proliferation through controlling the Wnt/*β*-Catenin signaling pathway.

## 1. Introduction

Lung cancer (LC) is considered the most common type of cancer. It is a heterogeneous disease that consists mainly of two subtypes, nonsmall cell lung cancer (NSCLC) and small cell lung cancer (SCLC), and remains the leading cause of death across the world [1]. The efficiency of current treatment options is hampered by pharmaceutical resistance, either inherent or acquired, as well as considerable side effects. Its return and spread to other parts of the body are also serious dangerous signs [2].

Mutations in genes and transmission of biological information play an important role in the tumor process [3–7]. Placenta-specific protein 8 (PLAC8) is a small 12.5-kDa protein that was initially screened and identified in mouse placental trophoblast cells [8] and was subsequently identified in plasmacytoid dendritic cells and giant trophoblasts [9, 10]. It is highly conserved and is expressed in plants, fungi, and animals [11–15]. During recent investigations, it has been shown that PLAC8 plays a vital role in the death of human lymphocytes as well as autophagy, adipose differentiation, pneumonia, and, most significantly, cancer [16–21].PLAC8 overexpression suppresses the development of liver cancer cells [22] but enhances colon cancer cell invasion and epithelial-mesenchymal transition in recognized PLAC8-related malignancies [23].Furthermore, PLAC8 overexpression is strongly associated with poor outcomes in patients with pancreatic, prostate, and renal cancer [24–28].

Previously, Novel et al. [29] confirmed that PLAC8 overexpression was related to the development of lung cancer suppressed by Kruppel-like factor 4 (KLF4) transcription. In another research [22], it was shown that overexpression of PLAC8 decreased the activity of liver cancer cells via inhibiting the Wnt/*β*-Catenin signaling pathway. In this retrospective analysis, we were able to establish that overexpression of PLAC8 increased the growth of lung cancer cells strongly. As far as we know, this is the first work to establish that the Wnt/*β*-Catenin signaling pathway (mediated by PLAC8) is involved in the growth of lung cancer cells. This route has the potential to become a novel therapy targeting lung cancer patients, with the goal of improving their outcomes.

## 2. Materials and Methods

### 2.1. Cell Culture and Treatments

Lung cancer cell lines (H838, A549, HCC 827, Calu-1, H322, H1975, H2347, H1299, SK-mes-1, and LLC) and BEAS-2B (human normal lung epithelial cells) were purchased from the Shanghai Cell Bank, Chinese Academy of Sciences. The cells were grown at 37 °C in a humidified atmosphere with 5% CO_2_ in Dulbecco's modified Eagle's medium (DMEM) supplemented with 10% fetal bovine serum (FBS), 100 IU/ml penicillin, and 100 *μ*g/ml streptomycin.

### 2.2. Animal Tumor Induction and Analysis

We adopted the approach provided by Kim et al. [30] as a point of reference. After incubating LLC and LLC-PLAC8 cells in a culture medium, the cells in the exponential growth phase were digested, harvested, washed, and adjusted to a concentration of 2.5 × 10^6^ cells/mL. Next, 200 *μ*l of cell suspension was injected into 6- to 8-week-old C57BL/6 J mice via the tail vein. The LLC group had 40 mice, whereas the LLC-plac8 group had 40 mice. The two groups were split into two subgroups (group 1 and group 2), each with 20 mice. Group 1 was executed, and tumor development was examined four weeks later, whereas group 2 was regularly examined and monitored for 50-day survival rate.

### 2.3. CCK8 Cell Viability Assay

Cells were seeded in 96-well plates at a density of 1.5 × 10^3^ cells/well. At each time point, 10 *μ*l CCK8 solution (CK04, Dojindo, JPN) was added directly to the test cells and incubated for 1 h at 37 °C. The absorbance was measured at 450 nm. The absorbance on days 1-7 was normalized to the absorbance on day 1, which was used as a control (100%). Each experiment was performed in triplicate.

### 2.4. Anchorage-Independent Colony Formation Assay

(24-) well plates were precoated with 0.8 ml of 0.5% (w/v) agar containing 10% (v/v) FBS as a bottom layer. The cell lines were suspended in 0.6 ml 0.3% (w/v) agar containing 10% (v/v) FBS and plated onto the bottom layer. Colony forming efficiency was determined for 2 to 3 weeks after plating and cultivation in humidified 5% CO_2_ atmosphere at 37 °C. The colonies were visualized after staining with 0.005% crystal violet

### 2.5. Colony Formation Assay

A total of 500/well cells were plated in 12-well plates and cultured for 10 days. The colonies were stained with 1% crystal violet stain for 1 min after fixation with 4% formaldehyde for 3 min. The colonies were counted, and the results are shown as the fold-change compared to the number of vector control cells.

### 2.6. Scratch Migration Assay

The spreading and migration capabilities of A549 cells were assessed using a scratch assay measuring the expansion of a cell population on a given surface. The cells were seeded into uncoated six-well tissue culture dishes at a density of 2.5 × 10^5^ cells and cultured in medium containing 10% FBS to nearly confluent cell monolayers, which were then scratched using 10 *μ*l sterile pipette tips. Any cellular debris was removed by washing with PBS. The wounded monolayers were then incubated in 10% FBS medium for 48 h. The wound area in a marked field of view was inspected at different time intervals subsequently until closure to determine the distance migrated by the cells. The scratch was photographed under a light microscope by a blinded investigator. The width of the scratch was measured at 0 h, 6 h, 12 h, 24 h, and at last 48 h after treatment to measure the distance traveled by the cells. The difference between the width of scratch at 0 h and at a given time point represented the distance migrated by the cells. The experiments were repeated in duplicate wells at least three times.

### 2.7. RNA Isolation and Real-Time PCR

Total RNA was extracted from cells with Trizol Reagent (#15596026, Invitrogen, USA) following the manufacturer's instructions. RNA concentration and purity were determined by measuring optical density at wavelengths of 260 and 280 nm using a standard spectrophotometer. cDNA was synthesized by reverse transcription reaction using strand cDNA synthesis kit (6210A, Takara, JNP). Real-time PCR was performed using the TB Green Premix Ex TaqTM (RR820A, Takara, JNP) on an ABI 7500 Real-Time PCR system machine (Applied Biosystems Inc., USA).The primers used were as follows: GAPDH forward, 5′-AGGTCGGAGTCAACGGATTTGGT-3′; GAPDH reverse, 5′-GTGCAGGA GGCATTGCTGATGAT-3′; PLAC8 forward, 5′-CTGTCTGTGTGGAACAAGC-3′; and PLAC8 reverse 5′- GAGGACAGCAAAGAGTTGCC-3′.

### 2.8. Immunohistochemical Analyses

Tissues were cut into sections 5 *μ*m thick, dewaxed with xylene, and the slides were washed in decreasing concentrations of ethanol (100%, 100%, 95%, and 75%) for 3 minutes each time. Immerse slides were washed in 4% paraformaldehyde in PBS for 5 minutes. They were washed 3 times for 5 min each in PBS (p1010, Solarbio, CHN). We used 0.01 mol/L citric acid antigen repair solution (22F00120, Dingguo, CHN) and thermal repair at 98 °C for 15 min to expose the antigen epitopes. It was treated for 12 min at room temperature with 3% H_2_O_2_ in PBS to block endogenous peroxidase activity. It was washed 3 times for 5 min each in PBS. For the primary antibody labeling, the slides were incubated with diluted rabbit polyclonal antibodies to PLAC8, Ki-67, *β*-Catenin, and PCNA at 4 °C overnight. The next day at room temperature for 1 hour, it was washed 3 times for 5 min each in PBS. Tissue sections were soaked with EnVision™ + System/HRP (DAB) (GK500710, Gene Tech, CHN). The tissues were then counterstained with hematoxylin and dehydrated with ethanol and xylene to prep for mounting. Brown staining in the nucleus was considered an indicator of protein expression and counted using a microscope (Olympus, JPN) at a magnification of ×200. For this purpose, five fields per slide were randomly selected by the viewer for evaluation.

### 2.9. Western Blot Analysis

Total protein was extracted from cell using RIPA buffer (#9806, CST, USA), and concentrations were measured using a BCA protein assay. The samples (50 *μ*g/sample) were separated by 12% SDS-PAGE (SD6014, BBI, CHN) and transferred onto a nitrocellulose filter membrane (#10401196, Whatman, USA). The membranes were blocked with skimmed milk with 5% and incubated overnight at 4 °C with the following primary antibodies: PLAC8 (ab122652, Abcam, USA), PCNA (#13110, CST, USA), CyclinD1 (#2978, CST, USA), C-myc (#5605, CST, USA), total-*β*-Catenin (#9587, CST, USA), unphosphorylated *β*-Catenin (#8814, CST, USA), GSK-3*β* (#12456, CST, USA), phosphorylated GSK-3*β* (#5558, CST, USA), AKT (#4685, CST, USA), phosphorylated AKT (#4060, CST, USA), and *β*-actin (#3700, CST, USA). The membranes were incubated with HRP conjugated secondary antibodies: goat anti-rabbit (AP132P, millipore, USA) and goat anti-mouse (AP124P, millipore, USA). The immunoreactive signals were visualized using an enhanced ECL chemiluminescence detection system (#7003, CST, USA).

### 2.10. In Vivo Animal Assay

BALB/c nude mice (6-week-old, weighing 20-30 g, female) were obtained from Shanghai Laboratory Animal Center, Chinese Academy of Sciences (Shanghai, China). The nude mice were fed in specific pathogen-free environment (temperature at 20-25 °C, humidity at 50-60%); all animals are cared for and used following the applicable international guidelines. The nude mice were injected with H838 cells with shCtrl vector and shPLAC8. The cell density was adjusted into 2.5 × 10^7^ cells/mL. The nude mice were partially disinfected and subcutaneously injected with 0.2 mL cell suspension at the root of thigh, and then general circumstance of the nude mice was observed, and the tumors were measured by a Vernier caliper every 3 d. After injected for 30 d, the nude mice were euthanized with their tumors extracted and paraffin embedded.

### 2.11. PLAC8 Overexpression and shRNA Knockdown in Cells

To generate PALC8-overexpressing cell lines, cell transfection was conducted following the manufacturer's guidance and transfected into A549 cells, LLC cells, and H 1975 cells. To knock down PALC8 in H838 cells and H322 cells, three different knockdown sequences and a blank vector were used to avoid any off-target effects. The sense sequences were as follows: PALC8-sh1, CAACTGAAATATGATGGATA; PALC8-sh2, AATGTTGTCCCTGAACTTAG; and PALC8-sh3, CTGATATGAATGAATGCTGT were cloned into the PLKO.1-puro shRNA vector and transfected into 293 T cells to produce lentivirus. The supernatants containing lentivirus were collected 48 h after transfection and then used to infect cells. The cells were selected with 1.5 *μ*g/ml puromycin for 2 weeks.

### 2.12. Luciferase Reporter Assay

Cells were seeded at a density of 2.5 × 10^5^ cells/well in 24-well plates. 0.2 *μ*g of firefly luciferase reporter plasmid, 0.002 *μ*g of renilla luciferase plasmid and 100 nM of hPLAC8, or hPLAC8 NC were transfected to each well using Lipofectamine 2000 (#11668-027, Invitrogen, USA). 48 h after transfection, the relative luciferase activity was confirmed following the Dual-Luciferase Reporter Assay Kit instructions (E1910, Promega, USA). The luciferase signal was detected at 560 nm for firefly luciferase and 465 nm for renilla luciferase, respectively. The ratio of firefly luciferase signal to renilla luciferase signal was calculated as relative luciferase activity.

### 2.13. Statistical Analysis

The results (mean value + standard deviation) of control (untreated) vs stimulated (treated) cell samples were analyzed using the Student's *t*-test for migration assays. Data analysis was performed with the SPSS 21.0 and GraphPad 5.0 software. Statistical significance was set at *p* < 0.05.

## 3. Results

### 3.1. Upregulation of PLAC8 Expression in Patients with Lung Cancer

**Figure 1:**
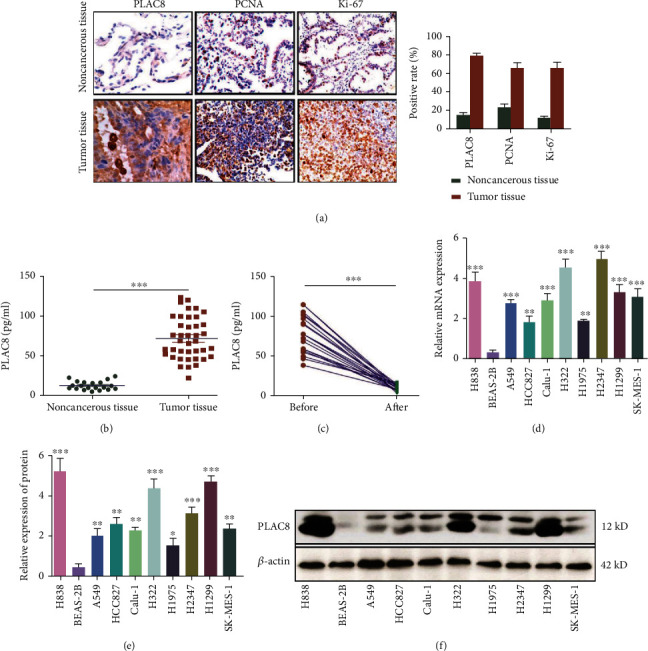
PLAC8 expression level in lung cancer patients and lung cancer cell lines. (a) PLAC8 expression level in lung cancer tissues and nontumor tissues (left: immunohistochemistry; right: statistical analysis). (b) PLAC8 expression level in serum of lung cancer patients and healthy people. (c) Expression of PLAC8 in serum of patients with lung cancer before and after operation. (d) PLAC8 mRNA expression level in lung cancer cell lines. (e, f) PLAC8 protein expression in lung cancer cell lines, ∗*p* < 0.05; ∗∗*p* < 0.01; ∗∗∗*p* < 0.001. All these cell biological function assays were triplicate.

Previous studies have confirmed that PLAC8 expression is increased in patients with lung cancer [29]. In this study, we performed immunohistochemistry (IHC) on the lung tissue from 68 patients with lung cancer (Figure 1(a)). The results showed that PLAC8 expression was significantly upregulated in the lung tissue from patients with lung cancer, which was consistent with our IHC results for PNCA and Ki-67, known as cancer-related genes in the lung tissue. The results suggested that PLAC8 expression in lung tissue was similar (significant upregulation) to that of other cancer-related genes. Next, we evaluated serum PLAC8 in 68 patients with lung cancer (Figure 1(b)); the levels were significantly higher than those in healthy controls. Moreover, serum PLAC8 in patients with lung cancer was significantly reduced after operation (Figure 1(c)).

We further analyzed the PLAC8 expression in different lung cancer cells (Figures 1(d), 1(e), and 1(f)). PLAC8 mRNA and protein expression levels were upregulated in H838, A549, HCC827, Calu-1, H322, H1975, H2347, H1299, and SK-MES-1 cells relative to the BEAS-2B control. Therefore, the undertaken study indicated that the expression of PLAC8 was upregulated in lung tissue and serum from patients with lung cancer.

### 3.2. Promotion of Cell Proliferation In Vivo and In Vitro by Overexpression of PLAC8

**Figure 2:**
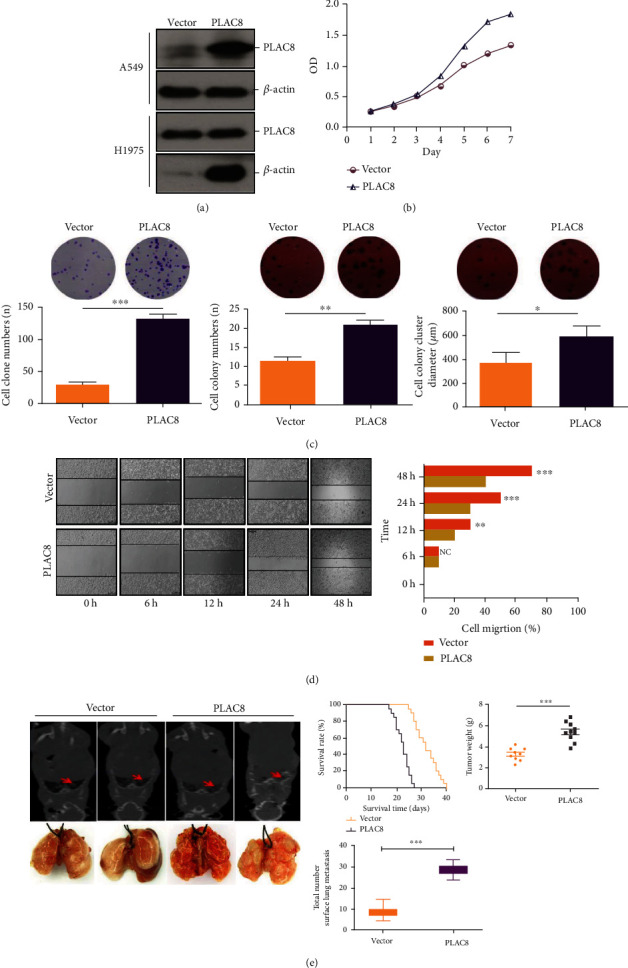
Overexpression of PLAC8 promotes the proliferation and migration of lung cancer in vitro and in vivo. (a) Protein level confirms overexpression of PLAC8. (b) Effect of PLAC8 overexpression on lung cancer cell proliferation. (c) Effect of PLAC8 overexpression on lung cancer cell clone proliferation (left: plate clone; right: soft agar clone). (d) Effect of PLAC8 overexpression on lung cancer cell migration. (e) Effect of PLAC8 overexpression on survival time (∗*p* < 0.05), tumor size (∗∗*p* < 0.01), and tumor metastasis (∗∗∗*p* < 0.001) of lung cancer mice. All these cell biological function assays were triplicate.

In the in vitro experiments, we transfected PLAC8 into lung cancer cell lines A549 and H 1579 using lentiviral vectors carrying the human PLAC8 gene. Firstly, we confirmed that the expression level of PLAC8 protein was significantly increased after transfection of both cell lines compared with the control group; Figure 2(a) indicated that the cells had been successfully transfected. Also, we found that PLAC8 overexpression promoted the proliferation of lung cancer cells (Figure 2(b)). Plate colony formation assays, soft agar colony formation assays, and scratch assays showed that PLAC8 overexpression promoted colony formation in vitro (Figures 2(c) and 2(d)). Following that, we used the tail vein to inject LLC and LLC-PLAC8 cells into C57BL/6 J mice. The findings revealed that PLAC8 overexpression dramatically decreased survival when compared to the control group, with statistically significant variations in tumor volume and weight between the two groups. Furthermore, more nodules were seen on the surface of the lungs in the PLAC8 overexpression group compared to the control group in this study (Figure 2(e)). It was determined from these findings that PLAC8 served as an oncogene in an experimental lung cancer model in mice.

### 3.3. Inhibition of Cell Viability and Proliferation In Vitro and In Vivo via the Downregulation of PLAC8 Expression

**Figure 3:**
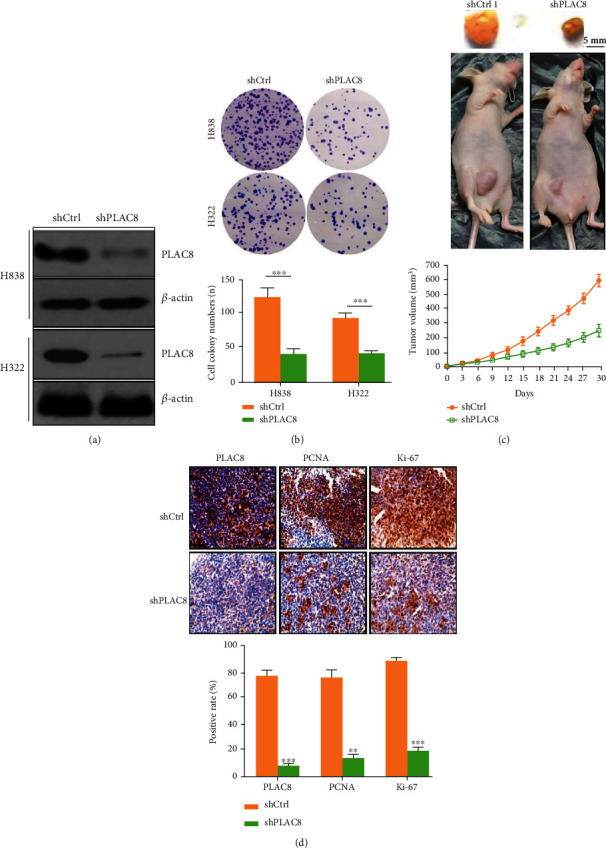
Downregulation of PLAC8 inhibits the proliferation of lung cancer in vitro and in vivo. (a) Protein levels confirm downregulation of PLAC8. (b) Downregulation of PLAC8 expression inhibits lung cancer cell clone proliferation. (c) PLAC8 downregulates inhibition of lung cancer proliferation in lung cancer mouse model. (d) Immunohistochemical detection of lung cancer in mice, ∗∗*p* < 0.01; ∗∗∗*p* < 0.001. All these cell biological function assays were triplicate.

We also investigated the effect of reduced PLAC8 gene regulation on the viability and proliferation of H838 and H322 lung cancer cell lines in vivo and in vitro. To inhibit the PLAC8 gene, we used a lentiviral vector carrying PLAC8-specific shRNA. The results showed that after transfection of H838 and H322 cells, the expression levels of mRNA and PLAC8 protein in the experimental group were significantly lower than those in the blinded control group (Figure 3(a)). IHC assays showed similar results. In plate colony formation assays, PLAC8 knockdown inhibited H322 and H1299 cell viability, cell proliferation, and growth (Figure 3(b)). In mice, the tumor grew significantly slower, and the tumor volume was significantly smaller at 30 days after implantation in the PLAC8 knockdown group than in the control group (Figure 3(c)). These results indicated that PLAC8 silencing inhibited lung cancer cell growth both in vitro and in vivo.

### 3.4. Regulation of the Wnt/*β*-Catenin Signaling Pathway in Lung Cancer Cells by PLAC8

**Figure 4:**
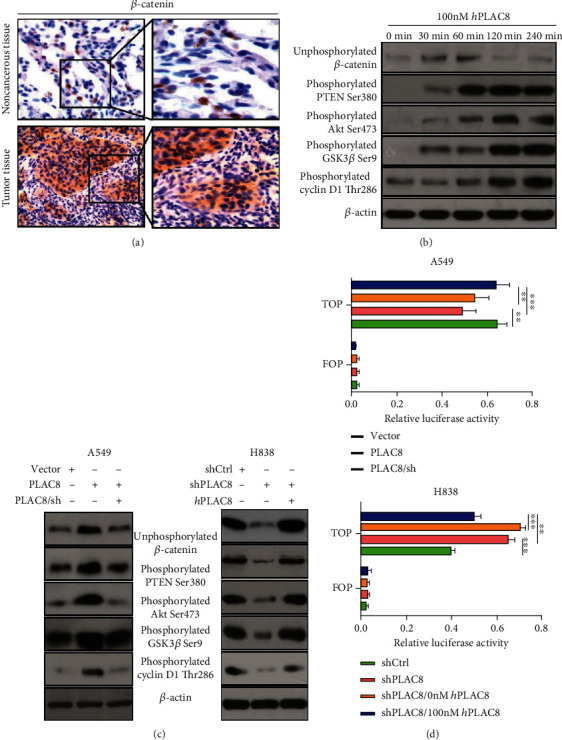
PLAC8 regulates lung cancer cell growth by regulating Wnt/*β*-Catenin signaling pathway. (a) Expression of p-Catenin in lung cancer tissues and nontumor tissues. (b) Lung cancer cell were treated by 100 ng/mL PLAC8 for the indicated times. (c) Effects of overexpression and downregulation of PLAC8 on Wnt/*β*-Catenin signaling pathway (Western blot). (d) Dual luciferase reporter gene analysis reveals that PLAC8 regulates Wnt/*β*-Catenin signaling pathway activity in A549 and H838 cells, ∗∗*p* < 0.01; ∗*p* < 0.001. All these cell biological function assays were triplicate.

We examined the expression of Wnt/*β*-Catenin signaling pathway components in A549 and H838 lung cancer cells following PLAC8 overexpression and PLAC8 silencing to learn more about PLAC8's regulatory route in vivo. IHC tests revealed that the PLAC8 overexpression group had more positive staining than the blank control group (Figure 4(a)), which further indicated that the PLAC8 overexpression group had higher levels of Wnt/*β*-Catenin signaling pathway components.

The expression of components of the Akt and GSK3 signaling pathways, as well as cyclin D1, increased with time has been shown by Western blot analysis (Figure 4(b)). The activity of the Wnt/*β*-Catenin signaling pathway was found to be significantly increased in H838 cells after PLAC8 overexpression, whereas it was found to be significantly decreased in A549 cells after PLAC8 silencing (Figures 4(c) and 4(d)) indicating that the ectopic expression of PLAC8 significantly enhanced *β*-Catenin activity, while the silencing of PLAC8 inhibited *β*-Catenin activity as reported by Zou et al. [22].

## 4. Discussion

Lung cancer is the leading cause of cancer-related deaths worldwide, accounting for 19% of all cancer-related deaths. Although many treatments are available for lung cancer, such as chemotherapy and surgery, however, the mortality to morbidity ratio associated with this cancer is still as high as 0.87 [31, 32]. PLAC8, also known as onzin, is a cysteine-rich protein that expresses itself in a particular tissue-specific manner. It was discovered for the first time in human dendritic cells. In addition to express itself in human oocytes and preimplantation embryos, it also governs the development of the placenta and embryos, which is a feature that has been preserved throughout evolution [33, 34]. PLAC8 is widely expressed in fungi, higher plants, and animals [12, 35, 36]. It plays a key role in regulating adipose differentiation, cell immunity, bone marrow proliferation and apoptosis, diabetes, adult-onset Still's disease, and the occurrence and development of cancer [16, 17, 20, 37–39]. Moreover, PLAC8 promotes apoptosis and cell proliferation in humans [19]. Current studies have shown that PLAC8 promotes cancer cell proliferation in the colon, pancreas, kidney, prostate, and lung tissue [25–27, 29, 30], while PLAC8 overexpression inhibits cancer cell growth in the liver tissue [22].

We initially investigated the expression of PLAC8 in human lung cancer tissue and serum from lung cancer patients and discovered that PLAC8 expression was considerably greater in lung cancer tissue and serum from lung cancer patients than in healthy controls. Additionally, overexpression of PLAC8 enhanced cell proliferation and malignancy in vitro and in vivo, similar with prior reports [29]. However, contrary to prior studies, this research found that PLAC8 was significantly expressed in the majority of lung cancer cell lines. Following that, we discovered that knocking down PLAC8 dramatically reduced tumor development in mice. These findings suggest that PLAC8 works as an oncogene, promoting the development of lung cancer. PLAC8 knockdown dramatically reduced tumor cell growth, indicating that PLAC8 might be a potential therapy target for lung cancer. To go further into the process, we examined the activity of the Wnt/*β*-Catenin pathway, PLAC8's regulatory route.

Cell proliferation, differentiation, and organ development are all aided by the Wnt/*β*-Catenin signaling pathway. The incidence and progression of lung cancer are linked to abnormal activation of this system. DNA binding inhibitor 1 expression has been shown to be higher in colorectal cancer tissue in recent research. It keeps cancer cells stem-like via modulating PLAC8 expression and activating the Wnt/*β*-Catenin pathway [40]. In liver cancer, the abnormal expression of PLAC8 can activate the Wnt/*β*-Catenin pathway and promote the malignant proliferation of liver cancer cells [22]. Therefore, we surmise that the Wnt/*β*-Catenin pathway may be an important downstream pathway in lung cancer tissue that overexpresses PLCA8. We performed Western blot and IHC analyses and found that after PLAC8 overexpression, the expression of *β*-Catenin was increased at both the cellular level (lung cancer cells) and tissue level (lung cancer tissue). Quantitative reverse transcription-polymerase chain reaction (qRT-PCR) showed that the level of *β*-Catenin mRNA expression was also significantly upregulated.

An earlier study showed that PLAC8 contained a cysteine-rich domain (11 of the 33 amino acids) that could bind to Akt1 and Mdm2, resulting in Akt1 and Mdm2 phosphorylation and translocation into the nucleus to bind to the CCAAT/enhancer-binding protein *β* (C/EBP*β*) promoter, thereby promoting C/EBP*β* transcription and subsequently regulating brown adipose differentiation and body temperature [17]. There is a paucity of information available on the molecular mechanism of PLAC8 at this time. The investigations mentioned above looked into how PLAC8 gets into the nucleus and regulates transcription. Because both the mRNA and protein expression levels of *β*-Catenin were increased, we hypothesize that the protein might be the regulatory target of PLAC8. Using a dual luciferase reporter system, we discovered that PLAC8 binds to the promoter of *β*-Catenin, allowing it to control *β*-Catenin transcription and inhibiting its expression. To confirm whether *β*-Catenin is a downstream target of PLAC8, we performed IHC assays, qRT-PCR, and Western blots to analyze the mRNA and protein expression levels of *β*-Catenin and found that the expression levels were significantly increased after PLAC8 transfection. These results, along with the dual luciferase reporter system data, confirmed that *β*-Catenin was a downstream target and was positively regulated by PLAC8. Additional downstream pathways and targets, such as the Akt and GSK3*β* signaling pathways and cyclin D1, were also significantly upregulated over time. GSK is a major inhibitor of the Wnt/*β*-Catenin signaling pathway. It promotes Wnt/*β*-Catenin degradation through the phosphorylation of several conservative serine and threonine residues of *β*-Catenin. Akt activates *β*-Catenin transcription by phosphorylating GSK3*β*. In this study, Western blots showed that PLAC8 activated the Akt signaling pathway, which led to GSK3*β* phosphorylation and the suppression of GSK3*β* function, thereby upregulating the expression of unphosphorylated *β*-Catenin and activating the Wnt/*β*-Catenin signaling pathway. Under normal conditions, *β*-Catenin is mainly located in the cell membrane, with minimal free *β*-Catenin in the cytoplasm. However, when free *β*-Catenin accumulates in the cytoplasm and enters the nucleus, it initiates the transcription of cyclin D1, a downstream target, leading to excessive cell proliferation and cancer. Cyclin D1 is a cyclin that is expressed in G1 phase. It is an important modulator that promotes cell division to transit from G1 to S phase. Cyclin D1 is considered a proto-oncogene, and its sustained high expression will shorten the G1 phase, causing premature transition to the S phase and subsequently uncontrolled cell proliferation and cancer. This study showed that the expression of cyclin D1 was significantly increased after PLAC8 overexpression, suggesting that PLAC8 may also promote cancer by regulating cyclin D1.

We further used luciferase vectors to co-transfect PLAC8 and *β*-Catenin-Wnt or PLAC8 and *β*-Catenin-Mut into the lung cancer cell lines A549 and H838 and analyzed the dual luciferase activity in 48 hours. The results showed that in A549 and H838 cells transfected with PLAC8, the luciferase activity of the *β*-Catenin-Wnt reporter vector was significantly increased, with no significant change in the control group, suggesting that PLAC8 activates the Wnt/*β*-Catenin signaling pathway and can transduce signals through PTEN, Akt, GSK3*β*, and cyclin D1.

Therefore, this study shows that PLAC8 is overexpressed in human lung cancer tissue and serum. PLAC8 overexpression promotes cell proliferation and cancer in vivo and in vitro, while PLAC8 silencing significantly suppresses tumor growth. The main significance of the study is that we have found that the PLAC8 promotes lung cancer cell growth by activating the Wnt/*β*-Catenin signaling pathway. So, on the practical implementation part, we indicate that PLAC8 is a promising treatment target of lung cancer, which provides a basis and new ideas for targeted therapy for patients with lung cancer.

## Data Availability

The datasets supporting the conclusions of this article are included within the article.

## Abbreviations

PLAC8: Placenta-specific protein 8
KLF4: Kruppel-like factor 4
DMEM: Dulbecco's modified Eagle's medium
FBS: Fetal bovine serum
IHC: Immunohistochemistry
qRT-PCR: Quantitative reverse transcription-polymerase chain reaction
C/EBP*β*: CCAAT/enhancer-binding protein *β*
LC: Lung cancer
NSCLC: Non-small cell lung cancer
SCLC: Small cell lung cancer.
